# Discovery and Verification of Soybean Sprouting Selection Based on Quality Across Various Origins and Varieties: Varietal Effects on Sprouted Soybean Quality

**DOI:** 10.1002/fsn3.70016

**Published:** 2025-02-04

**Authors:** Minmin Li, Mengying Zhao, Jinfeng Shi, Yatao Huang, Long Li, Nuo Jin, Zhiqiang Kong, Jesus Simal‐Gandarad, Fengzhong Wang, Bei Fan, Hong Xie

**Affiliations:** ^1^ Institute of Food Science and Technology, Chinese Academy of Agricultural Sciences/Key Laboratory of Agro‐Products Quality and Safety Control in Storage and Transport Process/Laboratory of Agro‐Products Quality Safety Risk Assessment Ministry of Agriculture and Rural Affairs Beijing People's Republic of China; ^2^ State Key Laboratory for Biology of Plant Diseases and Insect Pests Institute of Plant Protection, Chinese Academy of Agricultural Sciences Beijing People's Republic of China; ^3^ Nutrition and Bromatology Group, Analytical Chemistry and Food Science Department, Faculty of Science Universidade de Vigo Ourense Spain; ^4^ College of Food Science Shenyang Agricultural University Shenyang China

**Keywords:** isoflavones, nutrient evaluation, quality variation, soybean sprouts

## Abstract

Research on soybean nutrition is crucial for Chinese agriculture and public health, as soybean sprouting enhances its nutritional value. However, the relationships between grain quality and soybean varieties are not yet fully understood. In this study, the quality of 49 soybean cultivars from 15 different origins was evaluated through multiple correlation analysis. Differences were observed based on their geographical origin, with Shandong Chongwen showing higher calcium and isoflavone levels than the others. A correlation was found between protein content and soybean daidzein (0.561***), as well as genistein (0.597***). Additionally, sprouting was shown to improve soybean nutrition. Among six high‐nutrient soybean varieties, increased levels of protein, minerals, and isoflavones were observed during germination. Phytic acid and oligosaccharide levels decreased by 64.7% and 94.5%, respectively, while saponin increased by 106.0%, indicating changes in antinutritional factors. The experiment establishes a clear relationship between soybean nutrients and the tested variables, highlighting the impact of germination on soybean quality and nutrient content.

## Graphical abstract

Different nutrients for soybeans may result from different cultivars and origins. The various nutritional contents are related, especially in terms of isoflavone. The nutritional composition of the soybean may change during germination, and the antinutritional component present throughout the sprouting process may be eliminated.

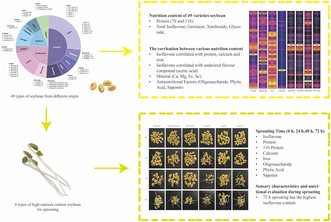

## 1 Introduction

Soybean is one of the most widely cultivated food and oil crops globally, with a history of over 4000 years of cultivation originating in China. As a result, soybeans are cultivated over a vast area, encompassing tens of thousands of varieties (Ghosh et al. 2021; He et al. 2023). Soybeans are highly valued for their macronutrient and biologically active compounds, including amino acids, unsaturated fatty acids, vitamins, and minerals. These components not only contribute to their high nutritional value but also play a significant role in preventing chronic diseases such as cancer, obesity, and cardiovascular disorders (Kim, Kim, and Yang 2021; Gebregziabher et al. 2022; Kusumah and Gonzalez de Mejia 2022).

Among the many processing methods for soybeans, sprouting has garnered attention for its ability to enhance nutritional value through metabolic changes during germination. Tofu made from sprouted soybeans, for instance, is considered nutritionally superior and has reduced legume aroma compared to traditional tofu, making it a healthy and popular food choice (Zinia et al. 2022; Pradhan et al. 2023). Sprouting is also a sustainable and accessible method that improves the nutritional quality of legumes. Numerous studies have explored methods to enhance soybean germination, such as exogenous plant hormones, chemical and physical treatments, and combined techniques like ultrasound and ascorbic acid treatments, which improve physicochemical characteristics and microbial safety (Hu et al. 2023; Shomodder et al. 2023; Wu et al. 2023).

In addition to processing methods, soybean varieties and their geographical origins significantly impact their nutritional composition. Soybeans in China are broadly categorized into northern, northeastern, and southern regions, with differences in soil conditions, climate, and field management practices affecting their growth and nutrient content (Xu et al. 2020; Malhi, Kaur, and Kaushik 2021). For instance, the northeastern region produces spring soybeans like Heidou and Jiyu, while southern regions focus on summer and autumn varieties such as Guichun and Guixia, known for their adaptability and nutritional quality. Geographical factors like soil pH and temperature also influence sugar, isoflavones, and mineral content (Malone et al. 2023; Roriz et al. 2023; Sattar et al. 2023).

Sprouting, a crucial processing method for soybeans, has been extensively studied for its effects on nutritional composition, particularly across different varieties and origins. However, few studies have specifically focused on the variations in nutrient types as a primary area of investigation. Given the vast diversity of soybean varieties worldwide, it is necessary to conduct research that incorporates soybeans from various origins with distinct compositions. Such variety‐specific research is critical to exclude the influence of individual soybean varieties on sprouting outcomes. In this study, 49 soybean varieties from northern, northeastern, and southern regions of China, as well as from overseas, were selected as research samples. The nutritional composition of these varieties, including protein fractions (7S and 11S), minerals (Ca, Mg, Fe, and Se), and isoflavones (genistein, xanthoside, and glycoside), was analyzed. Correlation among these components, as well as between isoflavones and their subcomponents, were examined. High‐nutritional‐value varieties were selected for further sprouting experiments to investigate the impact of sprouting on nutrient content. Six specific high‐quality soybean varieties were identified from the initial 49 samples. These were used to assess changes in various components, including isoflavones, proteins, iron, calcium, phytic acid, and oligosaccharides, under standardized sprouting conditions. Additionally, visual variations in soybean sprouts over time were documented. This research on the nutritional composition of soybeans and its transformation during processing provides valuable insights into global soybean agriculture and its potential applications in the food industry.

## 2 Materials and Methods

### 2.1 Materials and Chemicals

As briefly introduced in Figure S1, the 49 soybean varieties used in this study are mainly from 15 regions of origin, including northern China, northeastern China, southern China, and overseas regions. These soybeans were chosen based on their characteristics and their widespread cultivation in these regions. They were provided by the soybean industry system experimental stations in each province and city and stored at the Institute of Agricultural Products Processing, Chinese Academy of Agricultural Sciences. Every one of the 49 soybean cultivars was ground into soybean powder, with each sample weighing 20 g. The powder was then sieved through a 60‐mesh sieve and stored in sealed containers at −20°C.

Chemical reagents used for evaluating the quality of soybeans and sprouts were as follows: acetonitrile, methanol, and acetic acid (GC) from Merck, USA; soy isoflavones standard, phytic acid standard, soy saponin standard, and soybean oligosaccharide standard (not less than 98%) from Yuanye; sulfuric acid (GR) from Sinopharm Group Chemical Reagent Beijing Co.; copper sulfate, sodium hydroxide, sodium acetate, and 37% formaldehyde (GR) from Beijing Chemical Factory; sodium citrate, phenol, and ninhydrin buffer (GR) from Aladdin; hydrochloric acid (GR) from Shenzhen Bolinda Technology Co.; and Soybean 7S and 11S ELISA Kit from the Chincheng Bio.

### 2.2 Soybean Sprouting

To ensure distinct differences in the experiment, six specific soybean varieties were selected for the sprouting evaluation experiment, namely, Guixia No. 7, Mengdou 359, Shandong Chongwen‐Argentina, Fendou 93, Liaodou 32, and Brazilian soybeans, all known for their high nutritional content. These six soybean varieties were used for germination to analyze the changes in nutrient levels during sprouting. The soybeans were soaked in a 0.1% sodium hypochlorite solution for 5–7 h. Then, the water was filtered out, and the soybeans were spread out in an incubator with double gauze. The incubator sprouting temperature was 25°C, the relative humidity was 75%, and the sprouting cycle lasted for 72 h. Every 10 h, water was sprayed for 3 s. The growth stages of soybean sprouts were recorded and analyzed at 24, 48, 72, and 96 h.

### 2.3 Determination of Isoflavone Content

By utilizing the Agilent 1200 high‐performance liquid chromatography (HPLC) system, we conducted a comprehensive analysis of soybean samples. The process commenced by placing 50 g of the sample into a 250‐mL triangular flask, which was then firmly sealed. Precisely, 90 mL of 90% methanol was added to initiate the extraction process, conducted via ultrasonic agitation at 60°C for 30 min. Subsequent centrifugation at 10^5^ rpm for 10 min facilitated the separation of the supernatant from the residue. The supernatant underwent further treatment by supplementing it with an additional 60 mL of the 90% methanol solution to ensure thorough extraction. By merging both the initial supernatant and the supplementary extraction into a 250‐mL concentration flask, we meticulously reduced the volume to approximately 40 mL using a rotary evaporator, thereby enhancing the sample's concentration. This concentrated extract was then carefully transferred to a 50‐mL volumetric flask, ensuring rinsing using 10% methanol to prevent any material loss and achieving precise adjustment of the volume to the mark. For subsequent analysis, 1.0 mL of the prepared extract was filtered through a 0.45 μm membrane to eliminate any particulate matter that might interfere with HPLC analysis. This filtered extract was then poised for analysis on the HPLC machine, presenting an opportunity to gain insights into the composition of the soybean samples.

### 2.4 Determination of Protein Content

Modified in accordance with GB 5009.5–2016 “National Standard for Food Safety: Determination of Protein in Food,” (Zheng 2021a) the sample (normal and sprouted) was completely digested with sulfuric acid; neutralized with distilled water, p‐nitrophenol, and sodium hydroxide; diluted with water; and coupled to the standard solution. The concentration was calculated using the linear equation. In performing soy globulin ELISA, a microtiter plate was coated with purified soybean 7S and 11S globulins (GLB7s and GLB11s) capture antibodies to create a solid‐phase antibody complex. Soybean GLB7s and GLB11s were progressively added to the coated wells. This was followed by conjugation with an HRP‐labeled detection antibody to form antibody–antigen–enzymatic antibody complexes, which were then subjected to color development using the substrate TMB. The absorbance (OD) of the samples was measured at 450 nm using a microplate marker, and the concentration of soybean GLB7s and GLB11s was estimated based on the standard curve.

### 2.5 Determination of Mineral Content

Following the guidelines outlined in GB 5009.268–2016 “National Standard for Food Safety: Determination of Multiple Elements in Foods,” (Zheng 2021b) selenium, iron, magnesium, and calcium content in soybeans was analyzed by inductively coupled plasma mass spectrometry (ICP‐MS). Quantification was achieved using the external standard method, where the intensity of the mass spectral signal of the element under investigation correlates directly with its concentration. To commence the digestion process, 0.3 ± 0.001 g of solid sample was precisely weighed and transferred into a digestion tube. Subsequently, 10 mL of nitric acid was added to the tube. The digestion tube was securely capped, and the contents were subjected to the standard process of microwave digestion. Upon completion, the tube was allowed to cool, and the lid was removed cautiously, followed by a gentle rinse with a small amount of water. The digested solution was then heated at 100°C for 30 min, either on a temperature‐controlled hot plate or within an ultrasonic water bath, to ensure thorough dissolution and homogenization of the sample. This meticulous approach guaranteed optimal conditions for the subsequent analysis of the elemental composition of the soybean samples (normal and sprouted) using ICP‐MS.

### 2.6 Determination of Antinutritional Factor Content

In this research, an HPLC–tandem mass spectrometry (HPLC–MS/MS) approach was employed to rapidly quantify antinutritional components in soybeans (normal and sprouted), including saponins, phytic acid, and three specific oligosaccharides. The accuracy of the method was assessed by determining the linear equation, coefficient of determination [R (Bi et al. 2022)], the limit of quantification, the limit of detection, and the relative standard deviation. These parameters were calculated to ensure the precision and reliability of the analytical approach for determining the levels of antinutritional components in soybeans.

### 2.7 Statistical Analysis

All experiments were performed three times, and Microsoft Excel statistical software was used to evaluate statistical significance. ANOVA was used to analyze the values, with significance defined at the 95% confidence level. Figures were generated using GraphPad Prism 9.

## 3 Results and Discussion

### 3.1 Protein Content in Varieties and Origins

Soybean has the highest protein content among food crops compared with rice, maize, and wheat, making it an optimal choice for replacing meat with high‐quality plant‐based protein (Zhao et al. 2022). Soybean is an exceptional and well‐liked source of plant‐based protein, typically containing between 35% and 42% protein. Protein‐rich cultivars reach 45% or more, making them high‐quality vegetable proteins (Qin, Wang, and Luo 2022). As a result of the widespread cultivation of the soybean plant, thousands of nutritionally distinct varieties exist. To advance agriculture, it is necessary to select high‐quality varieties from various origins. The protein content of soybean varieties should always be the most important factor in determining their quality. In the study, 49 varieties of soybeans from diverse geographic regions were chosen to compare based on their origins. The total protein content and comparison between 7S and 11S were determined in this section.

**FIGURE 1:**
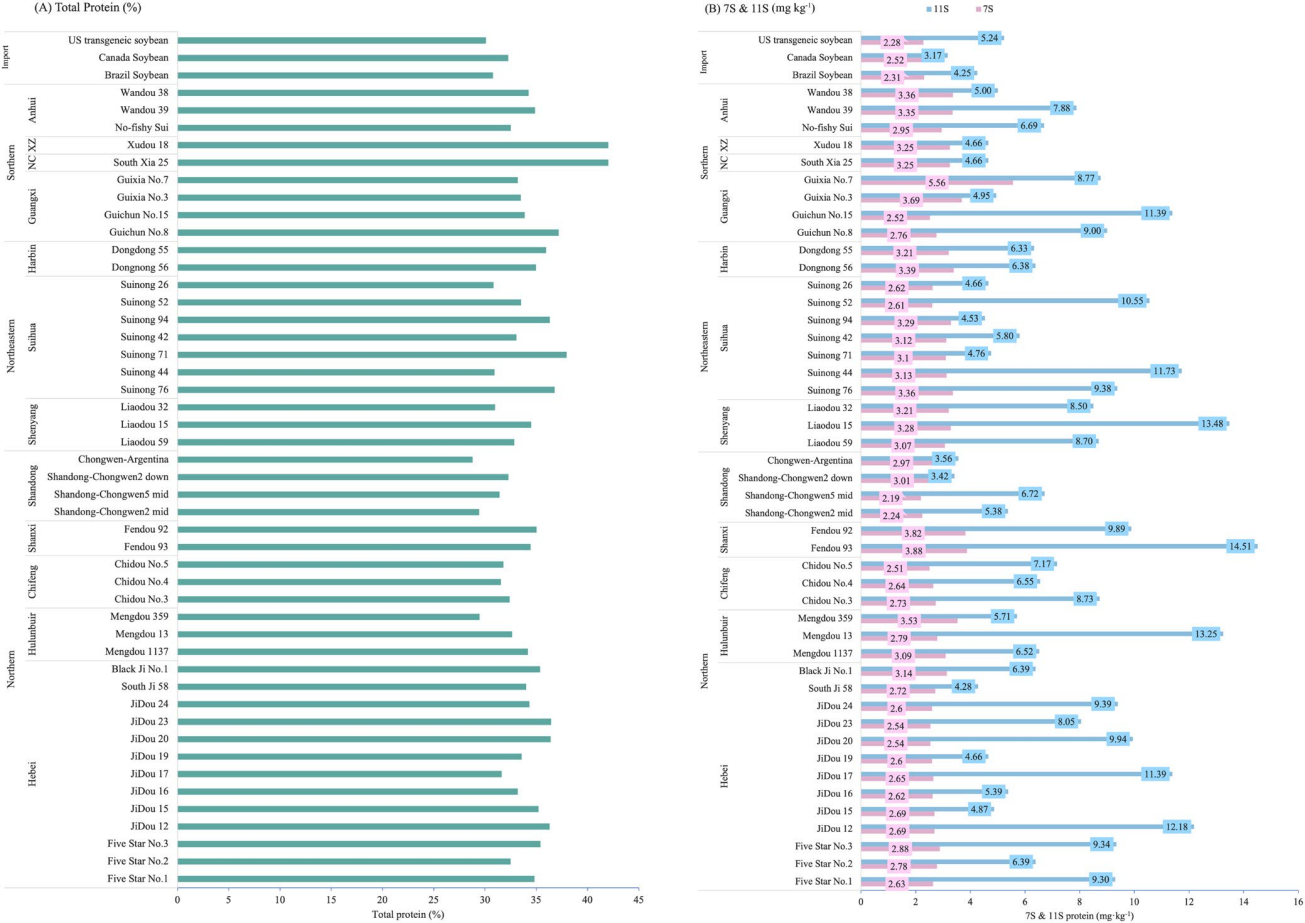
Comparison of total protein (%) and 7S and 11S protein (mg kg^−1^) in 49 types of soybean varieties, categorized by region of production. (A) Illustrates the total protein content as a percentage across different soybean varieties. (B) Presents the concentrations of 7S and 11S proteins (in mg/kg) for the same varieties, with 11S protein represented in blue and 7S protein in pink.

Protein content is the key indicator of the nutritional value of soy. Daily consumption of 25 g of soy protein is associated with a significant reduction in the risk of cardiovascular disease. This batch has an average total protein content of 34%, which represents a moderate level, as demonstrated in Figure 1. Among the production regions, both northern and imported varieties exhibit lower average contents at 33% and 31%, respectively. Moreover, the southern varieties had the highest average protein content in the study. The only variety with a protein concentration greater than 40% was the southern summer bean, with a 42% protein concentration for Xudou 18 and South Xia 18. This result is similar to previous study discussing the influence of the cultivation region. Soybeans are cultivated across a vast ecological region in China with varying environmental conditions, and the seed compositions of soybeans vary between regions. A distinct increasing trend in protein content from north to south in China is inversely correlated with latitude, which is evident (Song et al. 2023). In addition, high‐protein soybeans were primarily grown in the Yangtze River basin and the southwestern mountainous region, including central and eastern Sichuan, Chongqing, Hubei, northern and eastern Guizhou, northwestern Guangxi, central and eastern Zhejiang, and northwestern Yunnan. Our study corroborated this, showing that Guixia and Northeast Suinong had greater average protein levels than the norm, whereas Shandong Chongwen had lower‐than‐average protein levels. As a result, different cultivation regions can cause distinctive variations in protein composition.

As widely acknowledged, the 7S/11S proteins in soybeans are two commonly studied storage proteins, which generally account for about 70% of the total soybean protein. It has been reported that the total quantity of 7S/11S is greater in soybeans with higher protein content (Yang et al. 2020). Protein content, the content and proportion of 7S and 11S, and their subunit composition are closely associated with seed characteristics. These characteristics, such as gel‐forming and emulsifying properties, are important for food processing and affect the yield and quality of soy foods such as tofu and soymilk. Numerous studies have therefore examined the relationship between protein content and the content, proportion, and composition of these storage proteins (Cai et al. 2021).

The difference between 7S protein and 11S protein in this batch was relatively small, whereas the difference in 11S protein was more apparent in Figure 1. The differences in structure and amino acid composition of the two proteins contribute to the varying composition of their contents in soybeans. Southern soybeans exhibit a slightly higher average level of total protein and 7S protein compared with other soybeans, whereas imported soybeans have a lower protein content and vastly different protein content compared with other soybeans, particularly Canadian soybeans. Across all soybean types, the 11S protein content exceeds the 7S protein level, with Fendou 93 having the greatest 11S content and Guixia No. 7 having the highest 7S protein content. Although the previous results did not support the positive relationship between protein content and the level of 7S/11S or 11S, they did demonstrate a compensatory rebalancing between 7S and 11S protein as well as a redistribution of globulin subunits within the storage proteins (Yang et al. 2016).

The primary components of soybean storage proteins are 7S and 11S, whose functional qualities vary due to differences in amino acid compositions and structures, and whose interactions have a significant effect on the functional properties of soybean proteins (Zhu et al. 2023). The protein concentration of imported soy is lower. Shandong Chongwen has the lowest 7S and 11S contents among Chinese samples and is 40.6% below the average for 11S, compared to northeastern soybeans. In the four production regions, the northeastern region has the maximum 11S content, whereas the south has the highest average 7S protein content. Thus, the variations in 7S and 11S protein content can be attributed to differences in varieties and origins.

### 3.2 Mineral Content in Varieties and Origins

**FIGURE 2:**
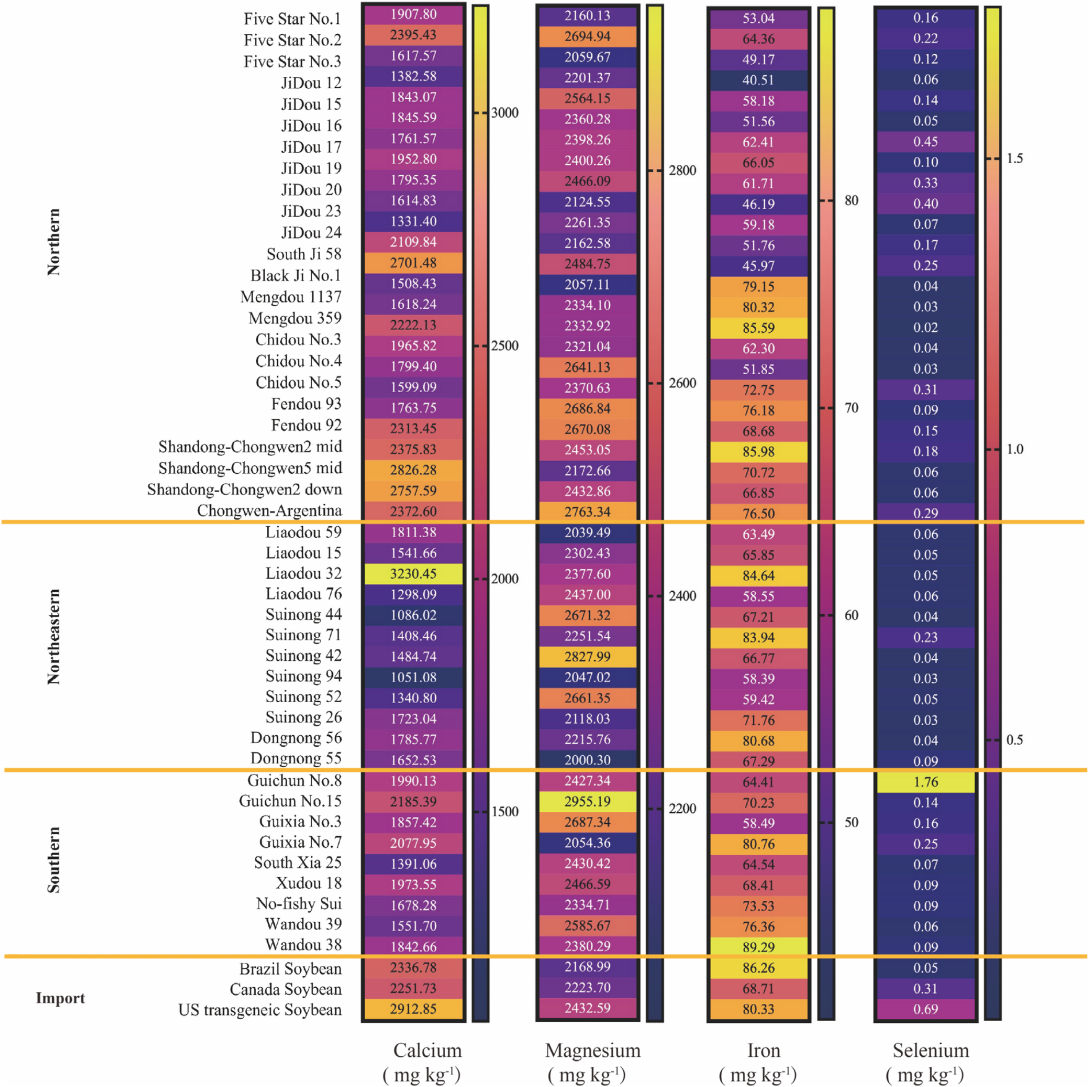
Contents of four mineral elements (calcium, magnesium, iron, and selenium) in various soybean varieties categorized by region (northern, northeastern, southern, and imported soybeans). The values are displayed in mg kg^−1^.

Soybeans contain numerous trace minerals beneficial to human health (Messina et al. 2022). As shown in Figure 2, calcium levels frequently exceeded 2200 mg kg^−1^ in soybean varieties with high isoflavone levels, such as the Shandong Chongwen cultivar, Brazilian soybean, and Mongolian soybean 359, as further discussed in Figure 4. This batch of soybeans had an average iron content of 68.0 mg kg^−1^. Eleven varieties contained more than 80 mg kg^−1^, including those with high isoflavone content and high 11S protein content, such as Liaozu 32 and Fenzu 93, which generally had lower selenium content. In contrast, Liaozu 32, Guixia No. 7, and Mengdou 359 exhibited superior magnesium content. As indicated by previous studies, the mineral content differences among soybean varieties are also related to the effect of salinity in the cultivation region. In many regions of the world, soil salinity is a significant barrier to legume cultivation. Salt‐tolerant species maintain high concentrations of calcium and potassium and low concentrations of sodium and chloride due to their ability to accumulate these elements in saline environments (Assaha et al. 2017). The unique soil conditions in Shandong are one of the primary reasons for the region's comparatively high mineral content. Thus, these specific regional conditions may affect the nutritional value. However, the impact is not limited to a single nutrient, as minerals may also affect other nutrients, such as isoflavones.

### 3.3 Isoflavone, Genistein, Xanthoside and Glycoside in Varieties and Origins

**FIGURE 3:**
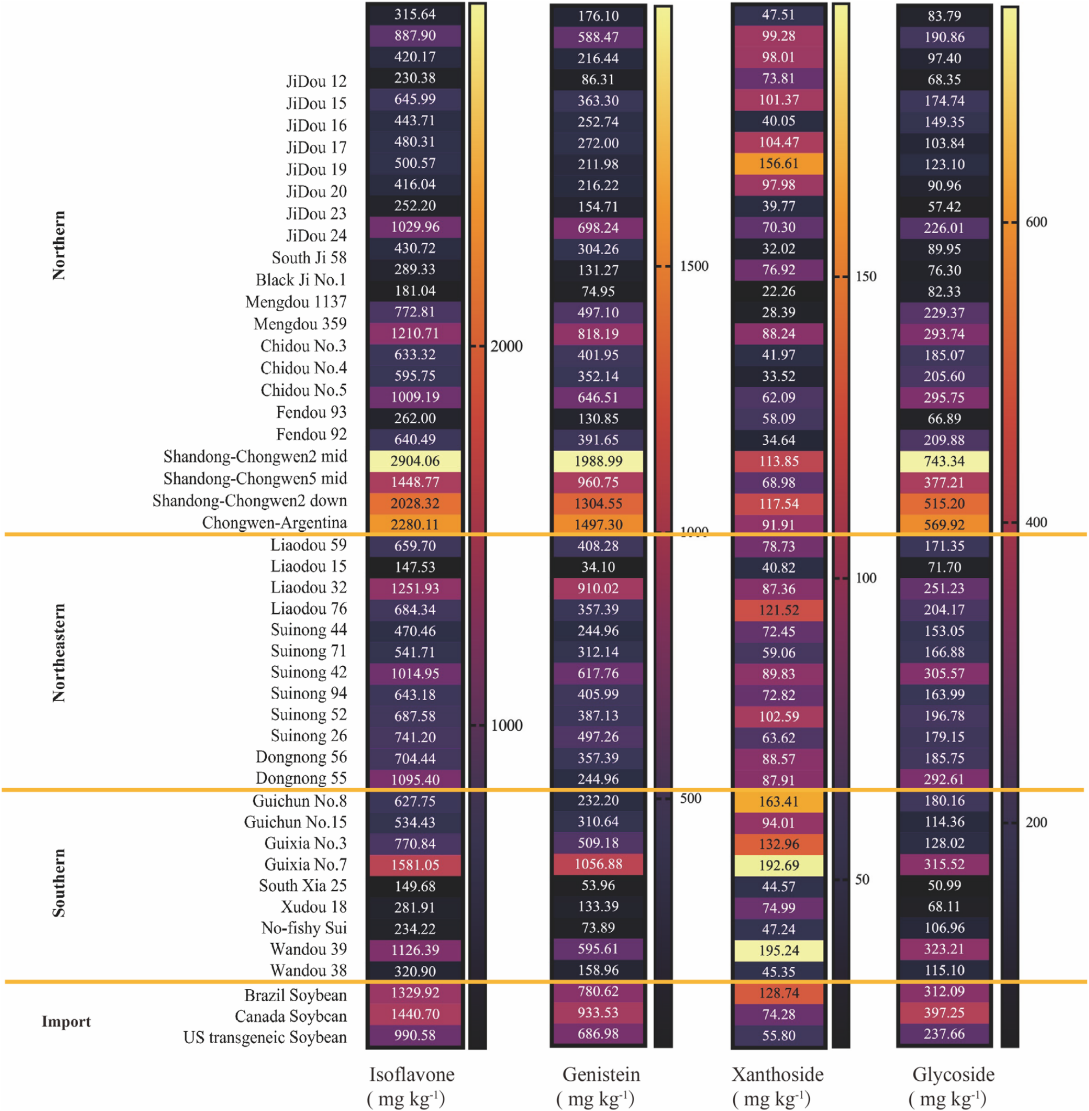
Comparison of nutritional content in 49 types of soybean varieties sorted by region of production: isoflavone, genistein, xanthoside, and glycoside. The regions include northern, northeastern, southern, and imported soybeans. The values are expressed in mg kg^−1^.

Soybeans contain isoflavones, including aglycones and glycosides (Kuligowski et al. 2022). Aglycones are present in lower concentrations than glycosides. Isoflavones, such as genistein, daidzein, and glycitein, along with their corresponding glycosides, account for approximately 50%, 40%, and 10% of the total isoflavone content of soybeans, respectively (Messina et al. 2021). Based on the classification of 49 types of soybean varieties by origin, we summarize the total isoflavone, genistein, xanthoside, and glycoside contents in Figure 3.

Isoflavones are a group of phytoestrogens derived from soybeans and their products. These phytoestrogens are secondary metabolites of the soybean growth process and affect various functions, including hormone secretion, metabolic biological activity, protein synthesis, and growth factor activity (Bi et al. 2022; Wang et al. 2022). Isoflavone concentrations typically range from 0.1% to 0.5%, with an average of 1000 to 1500 mg kg^−1^, but the majority of soybeans from Shandong exceed this range. In Hebei Jidou and Northeast China, the average soybean isoflavone concentration was less than 800 mg kg^−1^; however, varieties such as Jidou 24 and Liaodou 32 with concentrations exceeding 1000 mg kg^−1^ were exceptions. Liaodou 15, Mengdou 1137, and Nanxia 25 contained less than 200 mg kg^−1^ of isoflavones, ranking among the lowest varieties. According to research, the high isoflavone levels in Shandong soy are associated with the salinity of the region. The greater the isoflavone content of a plant's roots, the greater its nitrogen‐fixing activity and efficacy (Dong and Song 2020). Thus, to acclimatize to the conditions in Shandong, salt‐tolerant soybean varieties with a higher tolerance level are chosen for cultivation, which correlates with a high isoflavone content in Shandong soybean.

Genistein is the most prevalent isoflavone in soy, primarily present as glycoside bonds, and comprises more than 55% of the total isoflavones in most varieties. Shandong Chongwen 2‐mid contains the highest amount of genistein, at 1988.99 mg kg^−1^. In addition, most soybeans from Shandong have a higher genistein concentration than other varieties. Similar to the total isoflavone, Liaodou 15, Mengdou 1137, and Nanxia 25 had modest genistein concentrations.

Soybean xanthosides are frequently found as free glycosides and account for 10%–20% of the total soybean isoflavones, or approximately 100 mg kg^−1^. The concentration of isoflavone in the soy samples analyzed ranged from 40 to 200 mg kg^−1^. Wandou 39 had the maximum concentration of soybean xanthosides, at 195 mg kg^−1^, but its total isoflavone concentration was 1112 mg kg^−1^, and the proportion of soybean xanthin reached 17.6%. Guixia No. 7 had a high total isoflavone content of 192 mg·kg^−1^, or 12.1%. Other Guangxi varieties, including Guixia 3 and Guichun 8, had substantial levels of soybean isoflavones, exceeding 120 mg kg^−1^ and accounting for 17.2% and 25.4%, respectively, whereas Guichun 8 also had a relatively high proportion of soybean isoflavones.

Furthermore, soybean glycosides are one of the most prevalent isoflavone fractions. A comparison of their contents reveals that soybean glycosides from the Chongwen region of Shandong have a significant advantage, with an average content of 551 mg kg^−1^. In contrast, other varieties range from 100 to 300 mg kg^−1^, with the content in Shandong being approximately two to three times that of the other varieties. Shandong soybean daidzein was not significantly superior to those of other types, and unlike the majority of soybean glycosides, which are bonded glycosides, Shandong types contained fewer free glycosides. The soybean glycoside concentration of Guixia No. 7 and Wandou 39 remains high, measuring 102.7 and 195.2, respectively.

### 3.4 Correlation Between the Nutrient Compositions

Analyzing the nutritional content of soybeans may be conceivable to determine the relationship among each type of nutrient, select a high‐quality soybean breed, and provide evidence and a path for future cultivation.

The results are presented in Table 1, where ** denotes a highly significant correlation at the 0.01 level. The calculated data showed no significant correlation between the content of soybean xanthosides and the levels of isolated protein and mineral elements. However, total isoflavones, soybean glucosides, and genistein glucosides were highly correlated with the content of total protein, calcium (Ca), and iron (Fe) elements, except for soybean glycoside and 11S protein, for which the correlation was moderate.

**TABLE 1:** Correlation of the content of various substances in soybean.

Nutrition		Isoflavone	Daidzein	Soybean daidzein	Soybean genistein
Protein	Total protein	0.578**	0.039	0.561**	0.597**
	7S globulins	−0.22	0.273	0.076	0.018
	11S globulins	0.366**	0.078	0.346**	0.366**
Mineral	Calcium	0.480**	0.113	0.408**	0.501**
	Iron	0.411**	0.139	0.441**	0.440**
	Selenium	0.017	0.272	0.004	0.019
	Magnesium	0.169	0.033	0.125	0.195
Flavor compounds	N‐hexanal	−0.034	−0.03	−0.009	0.008
	I‐hexanol	0.181	−0.135	0.288	0.163
	Limonene	0.15	−0.206	−0.318	−0.392
	Acetic acid	−0.580**	0.177	−0.592**	−0.590**

** Denotes a highly significant correlation at the 0.01 level.

As shown in Figures 1 and 3, Shandong Chongwen exhibits low protein and high isoflavone levels. This observation is consistent with findings from investigations on cultivar BRS 258. Previous study has reported a weak negative correlation between protein content and individual isoflavones, which is also consistent with lower levels of minerals and bioactive peptides (Lee, Kim, and Hwang 2021; Shea et al. 2024). Thus, protein concentration influences the final distribution of nutrients and bioactive components in soybeans. The relationship among isoflavone, Ca, Fe, and 11S protein can serve as an indicator of the quality of soybean raw material and the sprouting procedure. Moreover, some studies have determined that genistein is bound in the interior portion of soybean glycinin (11S). This illustrates that there is a significant correlation between 11S protein and genistein (Smith et al. 2023).

The correlation between the isoflavone fractions in soybean and the primary flavor components in soybean is displayed. At the 95% confidence interval, the total soybean isoflavone, soybean glucoside, and genistein contents exhibited significant correlations (*p* < 0.05) with acetic acid content. This indicates that acetic acid is a major contributor to undesirable flavor in soybeans and a primary mechanism for the formation of a sour flavor in soybeans.

The selection of a larger number of species and indicators in this study was motivated by the desire to investigate the interplay between various factors and establish the extent to which substances influence one another. Although previous correlation studies between various substances and indicators have been conducted, these studies have the following limitations: The selection of a single species or a small number of species and the selection of a small number of indicators that do not constitute a system. Previous research has proposed a connection among protein, mineral elements, and isoflavones, which guides this investigation. The correlation coefficients between the three categories of isoflavones and the total protein content have been determined, except for the correlation between soybean xanthin and various elements, which was not statistically significant. Moreover, these three components have a significant relationship with the concentration of acetic acid, which is the primary mechanism for the development of an unpleasant flavor in soybeans.

Guixia No. 7, Mengdou 359, Shandong Chongwen, Fendou 93, Liaodou 32, and Brazilian soybeans were selected due to their high levels of isoflavones, micronutrients, and protein. These varieties demonstrate the correlation between nutrient profiles in the analysis. Using research on sprouting and processing conditions and quality variations could contribute to a better understanding of the nutrient variation principles and their correlation.

### 3.5 Nutrient Content and Quality Change in Sprouting Soybeans

According to the determination of nutritional content of the 49 types of soybean varieties, six types were chosen to evaluate the quantity and quality of the content in sprouting soybeans: Guixia No. 7, Mengdou 359, Chongwen‐Argentina, Fendou 93, Liaodou 32, and Brazilian soybeans. Isoflavone, protein, 11S protein, calcium, iron, oligosaccharide, phytic acid, and saponin were the key components of the nutritional analysis conducted during this sprouting experiment.

After 72 h of germination, the isoflavone concentration of Fendou 93 rose by 481.82 mg kg^−1^, representing nearly a 200% surge, making it the variety with the highest increase among the six examined. The trends in isoflavone content of Shandong Chongwen‐Argentina, Liaodou 32, and Fendou 93 closely followed the trends in total protein content. In a previous study yielding similar findings, total isoflavone concentrations in domestic and import cultivars ranged from 99 to 649.9 mg kg^−1^ and from 522.3 to 1287.7 mg kg^−1^, respectively. However, the isoflavone content of the different parts of soybean sprouts differed substantially. The sprout cap contained 69% genistein and 22% genistin, whereas the root contained 30% daidzin and 62% daidzein (Kim and Kim 2020). To demonstrate the quality variation in depth, a detailed analysis of isoflavone concentration in various parts of soybean sprouts during germination was conducted.

**FIGURE 4:**
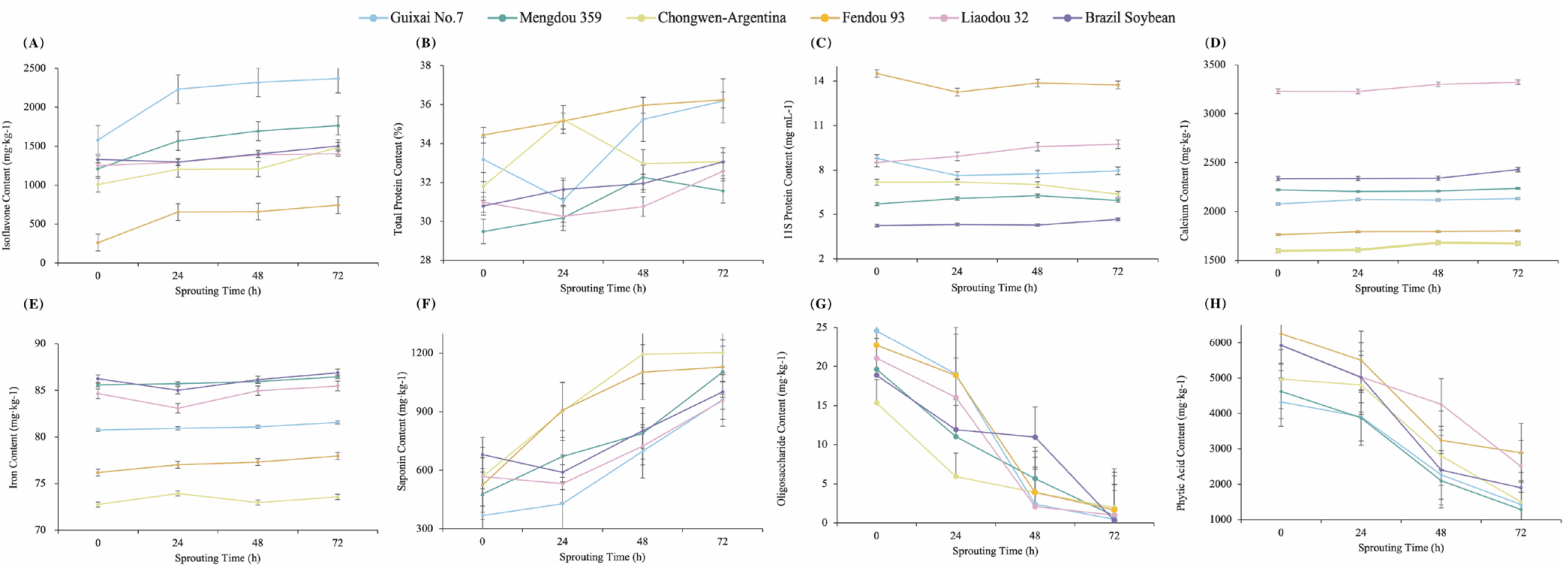
Changes of nutrition content during germination of different soybean varieties: (A) isoflavone; (B) protein; (C) 11S protein; (D) calcium; (E) iron; (F) oligosaccharide; (G) phytic acid; and (H) saponin. The sprouting progress is captured at four time points: 0 h, 24 h, 48 h, and 72 h. Except for protein (%), other values are expressed in mg kg^−1^.

Throughout the germination cycle, the total protein content of the six cultivars fluctuated. The initial concentration of protein was highest in Fendou 93 and lowest in Mengdou 359. The total protein content of Shandong Chongwen‐Argentine soybeans increased dramatically after 24 h of germination but returned to baseline after 72 h. The protein content of Guixia No. 7 decreased to 30% after 24 h of germination but increased to its peak level after 72 h. Over the course of 72 h, the protein content of other varieties, harvested at different times of the year, rose by between 1% and 4%. The temperature at which soybean seeds germinate has a substantial effect on their growth characteristics, nutrient composition, and secondary metabolites (Ma et al. 2022). Due to the optimal protein concentration in soybeans, the ideal temperature for this investigation is 25°C. This is consistent with the finding that trypsin inhibitor was highly expressed at 25°C and was identified as a secreted protein in soybean (Koo et al. 2015). Figure 4c illustrates the variations in 11S content that occur during germination in various soybean varieties. In comparison with the initial content, the final content of Mongolian bean 359, Liao bean 32, and the Brazilian soybean increased, whereas the 11S protein content of the other three varieties decreased, ranging from 0.7 to 1.3 mg kg^−1^, after sprouting.

In calcium analysis, all six varieties showed a general upward trend with moderate fluctuations, with increases of up to 100 mg kg^−1^. Guixia No. 7 had the highest initial and final calcium levels, while Brazilian soybeans exhibited the greatest increase at 92.7 mg kg^−1^. The study demonstrates that iron (Fe) levels increase during germination. Despite the fact that Fe levels increased from the beginning to the end of the germination cycle in all species, the average increase was less than 1.0 mg kg^−1^, which was not statistically significant. Notably, the Fe content of the Shandong Chongwen‐Argentina variety exhibits a trend similar to its total protein content, increasing sharply after 24 h and declining to a median level after 72 h. The same pattern holds true for Liaodou 32. This positive effect of germination on iron availability has previously been observed in soybeans, but germination has been shown to have a negative effect on calcium availability (Lu et al. 2023). Due to germination, this was not observed in the decrease in phytate content, although this was not observed in this study.

To further investigate the safety of sprouting, the levels of saponin, phytic acid, and oligosaccharides were analyzed in six varieties of soybeans. The dynamic content of phytic acid, oligosaccharides, and saponins in sprouts served as an indicator of the process's safety. The pattern of changes in the three types of antinutritional factors in soybeans over 72 h of germination shows that saponins accumulate, whereas phytic acid and oligosaccharide contents decrease by 64.7% and 94.5%, respectively. This pattern is consistent with previous studies on changes in antinutritional factors during germination. The saponin growth rates of the six soybean species ranged from 47.4% to 161.4%, with Guixia No. 7 exhibiting the highest growth rate. Shandong Chongwen‐Argentina bean sprouts had the highest saponin concentration at 1203 mg kg^−1^, whereas Brazilian soybeans initially had the highest saponin content but the lowest growth rate, with 1002 mg kg^−1^ in the final sprouts. As depicted, phytic acid and oligosaccharides in legumes are drastically reduced. The average decrease in oligosaccharide content was 94.5%, and Brazilian soybeans had the greatest reduction at 98.3%. Phytate is well known as a chelating agent that affects the bioavailability of minerals; consequently, mineral concentration is proportional to the phytic acid concentration. Food processes, such as soaking, germination, and fermentation, appear to be straightforward and nonchemical methods for reducing phytate and increasing the nutritional value of legumes (Zinia et al. 2022; Lu et al. 2023). Our research shows that extended germination time increases mineral content and decreases phytic acid content. Sprouting can therefore be an effective method for eliminating antinutritional factors and preserving nutritional value.

According to analyses of the dietary modifications that occur during sprouting, sprouting duration may be the factor that influences the nutrient composition. Consequently, it is necessary to compare the sensory properties and appearance of the germinated soybeans. Isoflavones, as the nutrient exhibiting the most significant variation, are also a key factor in determining the optimal conditions for germination.

**FIGURE 5:**
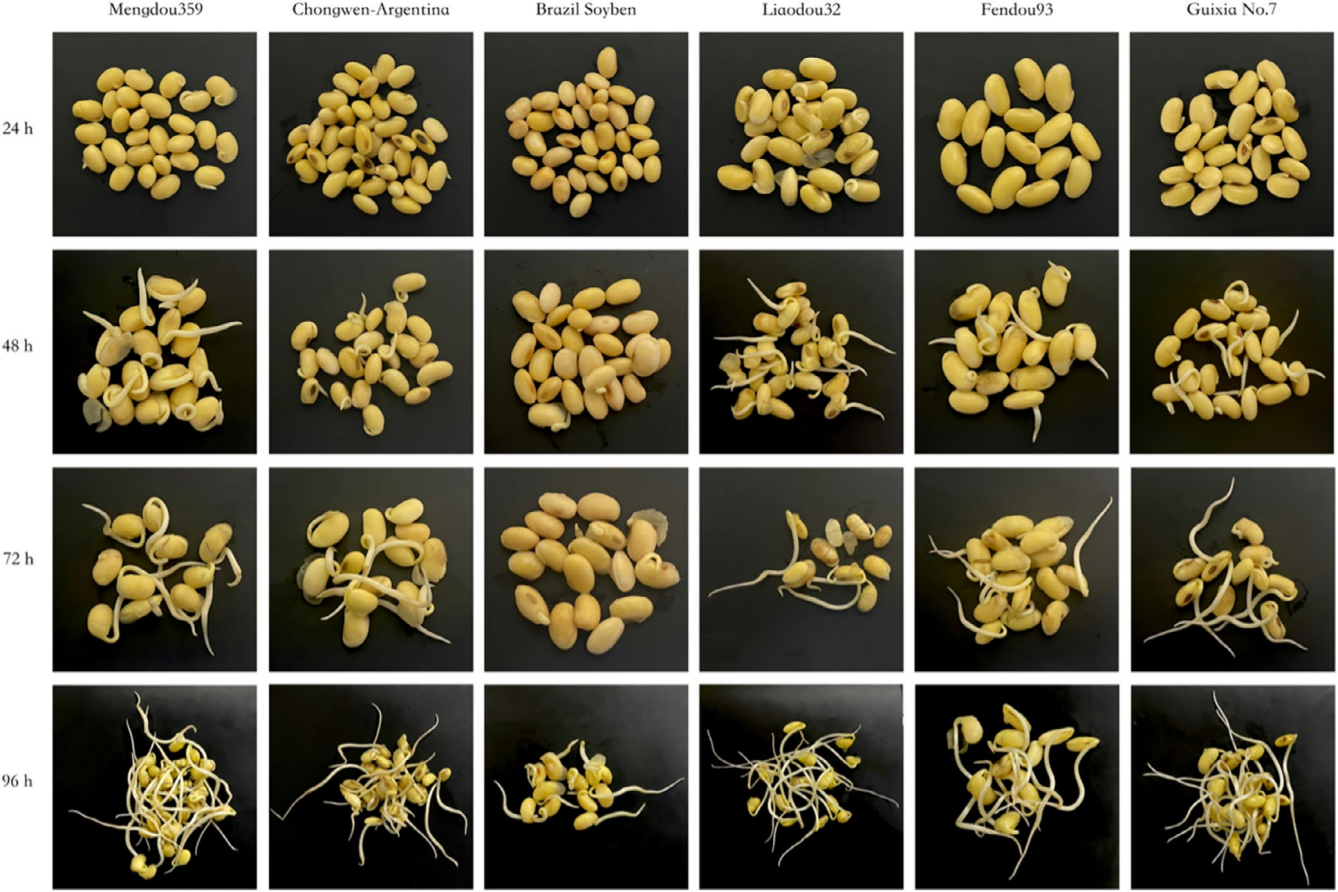
Appearance analyses of soybean sprouting of six varieties of soybean. The sprouting progress for six varieties of soybean is captured at four time points: 24 h, 48 h, 72 h, and 96 h.

In Table 2, the influence of sprouting duration on the quality of bean sprouts at an appropriate temperature (25°C) and humidity (85%) is examined. After 24 h of sprouting, no noticeable changes were observed in the soybeans, and the sprouts were typically less than 1.5 cm in length. After 48 h of germination, the length of the sprouts increased dramatically, and a faint bean aroma could be detected. At 72 h, the sprouts reached a length of nearly 5 cm, and the isoflavone concentration peaked. If the germination time surpassed 72 h, the bean sprouts developed a strong odor, and the sprout barbs grew significantly while the isoflavone content started to decrease. Figure 5 clearly illustrates the visual changes in the six varieties of soybean during the sprouting study.

**TABLE 2:** Impact of time on germination quality in soybean.

Sprouting time	24 h	48 h	72 h	Over 72 h
Corruption level	Basically nondeteriorating	Basically nondeteriorating	Some soybean darken	Significant spoilage and mold
Length of bean sprouts	1–2 cm	Around 3 cm	Over 5 cm	Massive manifestation of adhesion
Odor level	None	Slightly embryonic bean flavor	Distinctive embryonic bean flavor	Start stonk
Isoflavone content	2108 mg kg^−1^	2536 mg kg^−1^	3796 mg kg^−1^	Start deline

In the experiment examining the effect of germination on soybeans, the nutritional trend revealed that isoflavones and total protein increased significantly. Cultivars with the highest initial isoflavone content maintained the highest content after sprouting, although not the highest growth rate. During the germination process, the trend of total protein content closely mirrored that of isoflavones, whereas the trend of iron content was similar to that of total protein content, although less pronounced. Other substances, such as calcium and 11S protein, did not undergo significant changes during the germination process. Future studies may determine the correlation between the trends of these compounds and the content of other nutrients by conducting a large number of one‐time germination experiments. The significant correlation between total soybean isoflavones and soybean flavor suggests that sprouting could improve the flavor of soybeans by enhancing isoflavone and nutrient content. Phytic acid and oligosaccharide content decreased by 64.7% and 94.5%, respectively, whereas saponin content increased by an average of 106%. These results were generally consistent with the patterns described in the literature.

## 4 Conclusion

The isoflavone fraction, total and stored protein content, and mineral composition of soybeans were evaluated through physicochemical assays to compare the nutrient content of different varieties of soybeans. Varieties from various production regions differ in their chemical composition. The nutrient content of soybeans may be influenced by the environment in which they are grown, resulting in differences between domestic and imported varieties. Additional imported samples are required to fully evaluate the effect of cultivation regions in different countries. Nutritional differences were also assessed among several soybean cultivars. Variety is a key factor influencing nutrient composition, particularly isoflavones. According to the association between the various nutritional levels, isoflavones were strongly correlated with the total protein content, calcium and iron content, and 11S protein content and strongly inversely correlated with the acetic acid content. For the germination process, six varieties with higher overall quality across all nutrient levels were chosen. Among these, Guixia No. 7 from Guangxi exhibited the highest initial and accumulated isoflavone levels and mineral content. Sprouting was shown to enhance nutrient levels while reducing antinutritional factors, with an inverse relationship observed between the two during germination. Based on germination quality analysis, 72 h was identified as the optimal sprouting time, balancing nutritional value and appearance. This study provides new evidence supporting the nutritional improvement of soybean sprouts, offering reference data for evaluating sprout processing and enhancing the overall quality and safety of soybean‐based products.

## Author Contributions

**Minmin Li:** conceptualization (equal), formal analysis (equal), methodology (equal), project administration (equal), validation (equal), writing – review and editing (equal). **Mengying Zhao:** data curation (equal), formal analysis (equal), methodology (equal), validation (equal), writing – original draft (equal), writing – review and editing (equal). **Jinfeng Shi:** data curation (equal), formal analysis (equal), methodology (equal). **Yatao Huang:** writing – review and editing (equal). **Long Li:** investigation (equal), visualization (equal). **Nuo Jin:** methodology (equal). **Zhiqiang Kong:** conceptualization (equal), writing – review and editing (equal). **Jesus Simal‐Gandarad:** formal analysis (equal), writing – review and editing (equal). **Fengzhong Wang:** formal analysis (equal), methodology (equal), writing – review and editing (equal). **Bei Fan:** resources (equal), supervision (equal). **Hong Xie:** resources (equal), supervision (equal).

## Ethics Statement

This study does not involve any human or animal testing.

## Conflicts of Interest

The authors declare no conflicts of interest.

## Supporting information


**Table S1.** Validation of the detection method of soybean antinutritional factors.
**Figure S1.** Geographic origin information of the soybean varieties used in the study.

## Data Availability

Data sharing is not applicable to this article as no new data were created or analyzed in this study.
