# Correction to “Discovery and Verification of Soybean Sprouting Selection Based on Quality Across Various Origins and Varieties: Varietal Effects on Sprouted Soybean Quality”

**DOI:** 10.1002/fsn3.70193

**Published:** 2025-04-23

Li M , Zhao M , Shi J , Huang Y , Li L , Jin N , Kong Z , Simal‐Gandara J , Wang F , Fan B , Xie H . Discovery and Verification of Soybean Sprouting Selection Based on Quality Across Various Origins and Varieties: Varietal Effects on Sprouted Soybean Quality. Food Sci. & Nutr. 2025; 13:e70016. 10.1002/fsn3.70016 39906727 PMC11791410

The author name “Jesus Simal‐Gandarad” should be corrected to “Jesus Simal‐Gandara” (https://orcid.org/0000‐0001‐9215‐9737). The online version of this article has been corrected accordingly.

We apologize for this error.

