# Supplementary material for: Discovery and Verification of Soybean Sprouting Selection Based on Quality Across Various Origins and Varieties: Varietal Effects on Sprouted Soybean Quality
Source: Food Sci Nutr. 2025 Feb 4;13(2):e70016. doi: 10.1002/fsn3.70016 (PMC11791410; doi:10.1002/fsn3.70016)
Supplement: Supplementary file 1 — Table S1. Validation of the detection method of soybean antinutritional factors. Figure S1. Geographic origin information of the soybean varieties used in the study. [file FSN3-13-e70016-s001.docx]

**Supplementary materials**

**Table S1.** Validation of the detection method of soybean anti-nutritional factors

**Figure S1.** Geographic origin information of the soybean varieties used in the study.

**Table S1. Validation of the detection method of soybean anti-nutritional factors**

| Compound | R^2^ | Linear Equation | LOD (μg·kg^-1^) | LOQ (μg·kg^-1^) | RSD (%) |
| --- | --- | --- | --- | --- | --- |
| Sucrose | 0.9999 | Y=0.6172x-0.1838 | 0.69 | 2.32 | 12.81 |
| Cotton seed sugar | 0.9956 | Y=0.6207x-51.8364 | 0.43 | 1.42 | 8.42 |
| Stachyose | 0.9929 | Y=0.9862x-35.3089 | 0.32 | 1.06 | 6.03 |
| Saponin Aa | 0.9934 | Y=16.4906x-3.0912 | 0.2 | 0.68 | 6.48 |
| Saponin Ab | 0.9915 | Y=7.9213x-554.1460 | 0.05 | 0.16 | 7.41 |
| Saponin Ba | 0.9922 | Y=2.3112x-20.7535 | 0.076 | 0.25 | 18.84 |
| Saponin Bb | 0.9926 | Y=1.7036x-7.6247 | 0.11 | 0.36 | 10.91 |
| Phytic acid | 0.9938 | Y=0.7359x-36.1326 | 4.38 | 14.61 | 24.6 |

R^2^, Coefficient of Determination; LOD, Limit of Detection; LOQ, Limit of Quantification; RSD, Relative Standard Deviation.

**
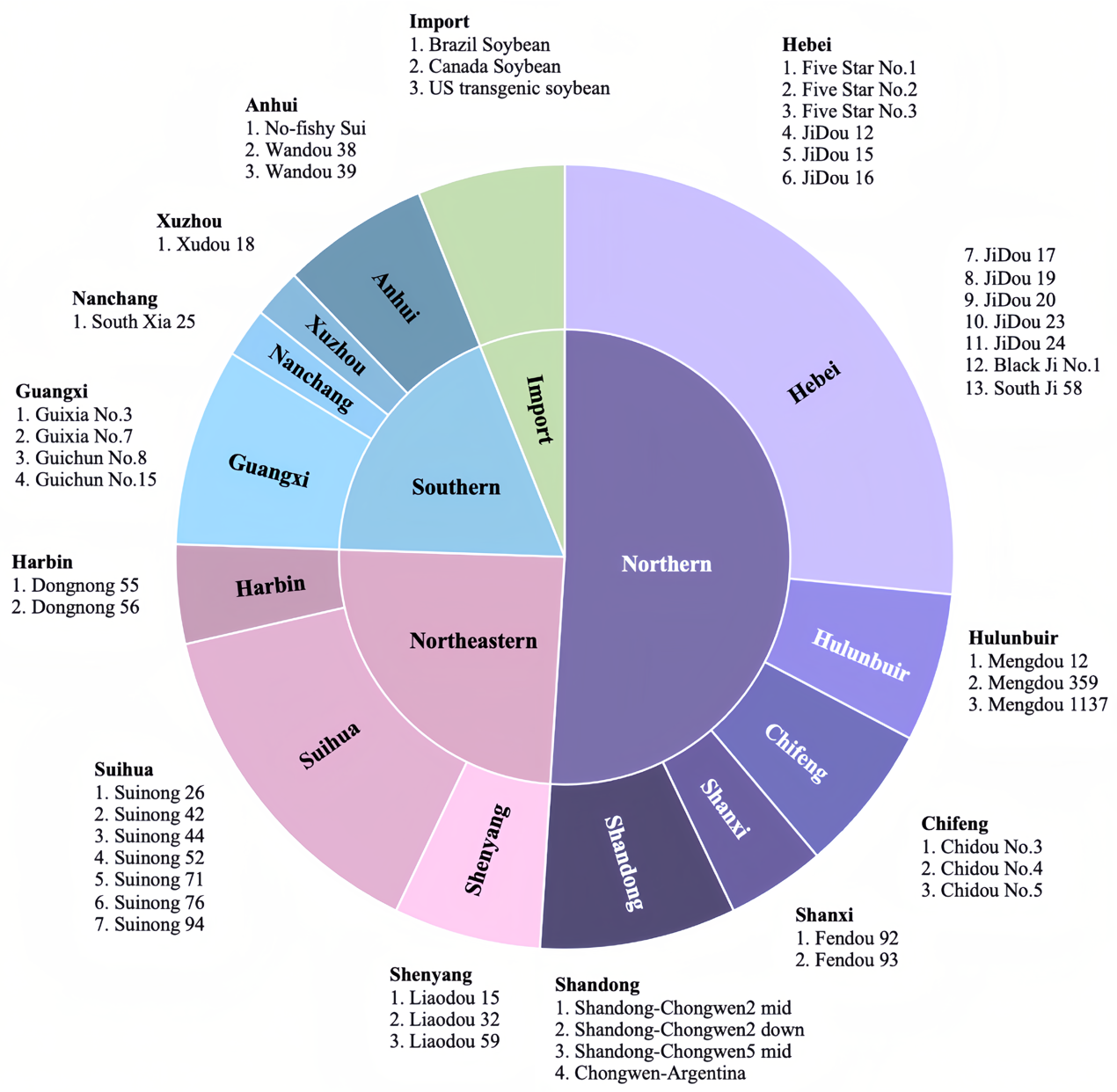
**

**Figure S1.** Geographic origin information of the soybean varieties used in the study. The chart categorizes soybeans by region, including Northern, Northeastern, Southern, and Imported varieties.
